# Clonal hematopoiesis activates procalcific pathways in macrophages and promotes aortic valve stenosis

**DOI:** 10.1172/JCI171634

**Published:** 2025-11-18

**Authors:** Wesley T. Abplanalp, Michael A. Raddatz, Bianca Schuhmacher, Silvia Mas-Peiro, María A. Zuriaga, Nuria Matesanz, José J. Fuster, Yash Pershad, Caitlyn Vlasschaert, Alexander J. Silver, Eric Farber-Eger, Yaomin Xu, Quinn S. Wells, Delara Shahidi, Sameen Fatima, Xiao Yang, Adwitiya A.P. Boruah, Akshay Ware, Maximilian Merten, Moritz von Scheidt, David John, Mariana Shumliakivska, Marion Muhly-Reinholz, Mariuca Vasa-Nicotera, Stefan Guenter, Michael R. Savona, Brian R. Lindman, Stefanie Dimmeler, Alexander G. Bick, Andreas M. Zeiher

**Affiliations:** 1 Institute for Cardiovascular Regeneration, Goethe University, Frankfurt, Germany.; 2 German Center for Cardiovascular Research (DZHK), Partner Site Frankfurt Rhine-Main, Berlin, Germany.; 3 Cardiopulmonary Institute, Goethe University, Frankfurt, Germany.; 4 Vanderbilt University School of Medicine, Nashville, Tennessee, USA.; 5 Division of Cardiology, Department of Medicine, UCLA, Los Angeles, California, USA.; 6 Division of Genetic Medicine, Department of Medicine, Vanderbilt University Medical Center, Nashville, Tennessee, USA.; 7 Department of Medicine 3 (Cardiology/Angiology), Goethe University, Frankfurt, Germany.; 8 Centro Nacional de Investigaciones Cardiovasculares (CNIC), Madrid, Spain.; 9 CIBER en Enfermedades Cardiovasculares (CIBER-CV), Madrid, Spain.; 10 Department of Medicine, Queen’s University, Kingston, Ontario, Canada.; 11 Division of Hematology and Oncology, Department of Medicine,; 12 Program in Cancer Biology,; 13 Division of Cardiovascular Medicine, Department of Medicine, and; 14 Department of Biostatistics, Vanderbilt University Medical Center, Nashville, Tennessee, USA.; 15 Department of Biomedical Informatics, Vanderbilt University, Nashville, Tennessee, USA.; 16 Center for Quantitative Sciences and; 17 Department of Pharmacology, Vanderbilt University Medical Center, Nashville, Tennessee, USA.; 18 German Heart Center, Technical University of Munich, Munich, Germany.; 19 Max Planck Institute for Heart and Lung Research, Bad Nauheim, Germany.; 20 Vanderbilt-Ingram Cancer Center, Nashville, Tennessee, USA.; 21 Center for Immunobiology and; 22 Structural Heart and Valve Center, Vanderbilt University Medical Center, Nashville, Tennessee, USA.

**Keywords:** Aging, Cardiology, Inflammation, Cardiovascular disease, Monocytes

## Abstract

Clonal hematopoiesis (CH) due to Tet methylcytosine dioxygenase 2 (*TET2)* driver mutations is associated with coronary heart disease and a worse prognosis for patients with aortic valve stenosis (AVS). However, it is unknown what role CH plays in the pathogenesis of AVS. In a meta-analysis of All of Us, BioVU, and the UK Biobank, patients with clonal hematopoiesis of indeterminate potential (CHIP) had an increased risk of AVS, with a higher risk among patients with *TET2* or *ASXL1* mutations. Single-cell RNA-Seq of immune cells from patients with AVS harboring *TET2* CH driver mutations revealed monocytes with heightened proinflammatory signatures and increased expression of procalcific paracrine signaling factors, most notably oncostatin M (OSM). Secreted factors from *TET2*-silenced macrophages increased in vitro calcium deposition by mesenchymal cells, which was ablated by OSM silencing. Atherosclerosis-prone low-density lipoprotein receptor–deficient (*Ldlr*^–/–^) mice receiving CH-mimicking *Tet2^−/−^* bone marrow transplants displayed greater calcium deposition in aortic valves. Together, these results demonstrate that monocytes with CH promote aortic valve calcification and that patients with CH are at increased risk of AVS.

## Abstract 2

Clonal hematopoiesis associated with mutations in TET2 associates with degenerative calcified aortic valve stenosis in humans and mice

## Graphical abstract

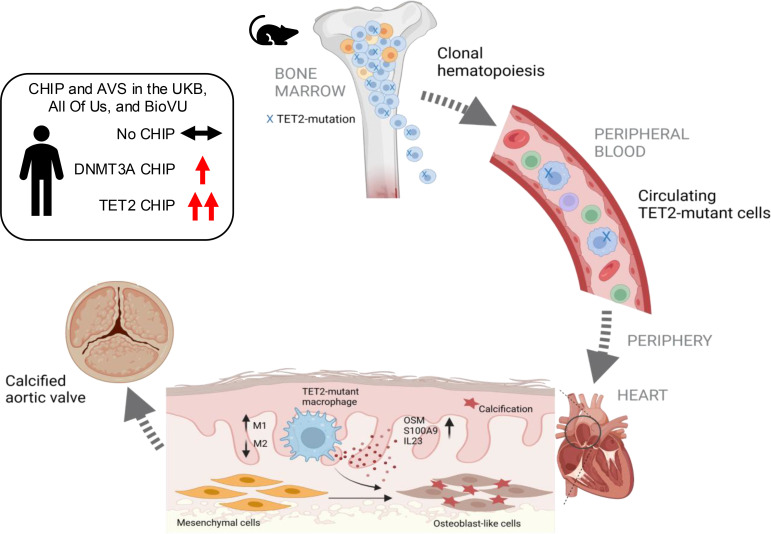

## Introduction

Calcific aortic valve disease is the most common acquired heart valve disease, affecting 2%–7% of those over 65 years of age in the western world and incurs a high burden of morbidity and mortality (1, 2). There is no medical therapy to halt progression to aortic valve stenosis (AVS), and the only treatment is aortic valve replacement in select patients (3). While aortic valve calcification is exacerbated by traditional cardiovascular risk factors and a proinflammatory milieu, risk prediction for progression to severe stenosis is still poor (2, 4). Thus, there is an unmet clinical need to understand the mechanisms of calcific aortic valve disease progression to identify possible therapeutic targets.

Clonal hematopoiesis (CH) is the age-associated acquisition of somatic mutations in hematopoietic stem cells, leading to clonal expansion. CH is associated with an increased risk of cardiovascular diseases (5), and CH driven by mutations in one of the most prevalent driver genes, Tet methylcytosine dioxygenase 2 (*TET2*), is associated with a proinflammatory gene signature in circulating immune cells in both mice and humans (6–8). Importantly, the prevalence of *TET2* CH driver mutations is markedly enriched in patients with severe calcified AVS and is associated with a worse prognosis even after removal of the stenotic aortic valve (9–11). However, the potential underlying mechanisms of AVS and whether CH is associated with incident AVS are presently unknown.

## Results

### Association of CH with aortic valve disease in BioVU, All of Us, and the UK Biobank.

**Figure 1:**
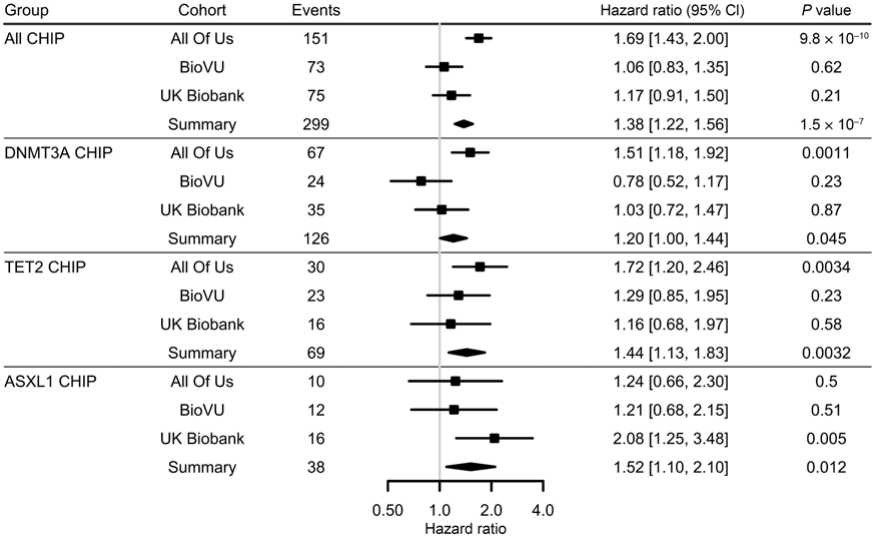
Association of CHIP with AVS in All of Us, BioVU, and UK Biobanks. HRs for all CHIP genes and AVS along with specific CHIP gene associations with AVS across three biobanks for all VAF. Horizontal lines indicate 95% CIs.

We first analyzed clonal hematopoiesis of indeterminate potential (CHIP) across All of Us (*n* = 275,679), BioVU (*n* = 160,012), and the UK Biobank (*n* = 450,687). CHIP was identified in 35,311 participants (~3.9%) using previously reported methods (12) (Supplemental Table 1). AVS was documented by International Classification of Diseases (ICD) diagnostic codes (Supplemental Table 2). As expected, CHIP carriers were older and had a higher prevalence of cardiovascular comorbidities (Table 1). In fully adjusted models across all cohorts, CHIP was associated with a significantly increased risk of AVS (HR = 1.38 [1.22–1.56], *P* = 1.5 × 10^–7^). Gene-pecific analyses revealed that *TET2* (HR = 1.44 [1.13–1.83], *P* = 0.0032) and additional sex combs like transcriptional regulator 1 (ASXL1), (HR = 1.52 [1.10–2.10], *P* = 0.012) mutations conferred the greatest risk, whereas DNA methyltransferase 3 alpha (DNMT3A) mutations were associated with a more modest effect (HR = 1.20 [1.00-1.44], *P* = 0.045) (Figure 1). Taken together, these results demonstrate that CHIP represents a risk factor for AVS across diverse populations.

### CH-associated gene expression signature in circulating myeloid cells from patients with severe calcific AVS.

**Figure 2:**
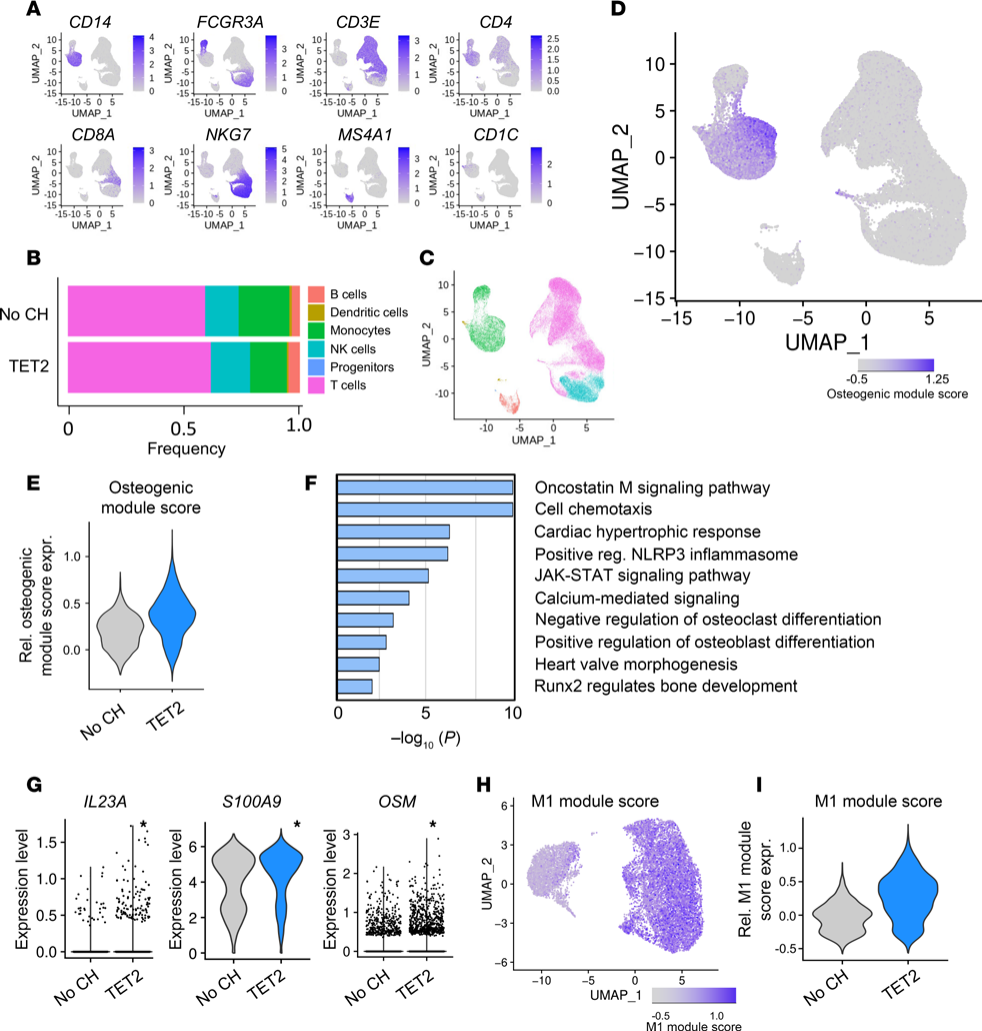
TET2-mutant carriers show increased expression of proinflammatory and procalcific mediators. (**A**) Uniform manifold approximation and projections (UMAPs) showing gene expression of cell-type–specific markers in scRNA-Seq of PBMCs from patients with and without CH mutations. (**B** and **C**) Relative abundance of cell types by mutation status (**B**) with annotations (**C**). (**D**) Expression of the osteogenic module score in PBMCs. (**E**) Expression of the osteogenic module score in human monocytes by violin plot. (**F**) Selection from the top GO terms called by upregulated genes in monocytes of patients harboring *TET2* mutations. (**G**) Expression of procalcific genes in patients’ monocytes. (**H** and **I**) Expression of the M1 macrophage module score in human monocytes by (**H**) feature plot and quantified in (**I**) a violin plot. Statistical significance in scRNA-Seq was determined using the FindMarkers function in Seurat. *P < 0.05.

Next, we sought to identify how CH mutations in the blood confer an increased risk of AVS. As myeloid cells have been previously shown to promote progression of AVS (13), we hypothesized that CH driver mutations may further accelerate this progression. We performed single-cell RNA-Seq (scRNA-Seq) on PBMCs obtained from AVS patients with and without mutations in the CH driver gene *TET2* (Supplemental Tables 3–5; supplemental material available online with this article; https://doi.org/10.1172/JCI171634DS1). Mean CH driver gene variant allele frequency was 17.9% (range: 2.6%–37.4%). Unsupervised clustering revealed 6 cell types in PBMCs, with no significant changes in cell type distribution with respect to the presence or absence of CH driver mutations (Figure 2, A–C, and Supplemental Figure 1). Among these cell types, monocytes demonstrated an upregulation of an osteogenic module score, which represents a composite expression of known paracrine mediators of calcification (Figure 2D). This module was more highly expressed in monocytes of patients harboring *TET2* mutations (Figure 2E), so we focused on a deeper characterization of circulating monocytes.

Monocytes of patients harboring *TET2* mutations had 4,320 upregulated genes compared with monocytes derived from patients without mutations. Gene Ontology (GO) terms of upregulated genes comprised inflammatory pathways including for oncostatin M (OSM) and osteoblastic differentiation (Figure 2F). Additionally, scRNA-Seq of monocytes from patients with CH documented increased expression of the procalcific, secreted inflammatory factors (e.g., OSM, S100A9, IL-23), which are known to support the mineralization and calcification of valvular and vascular cells (14, 15) (Figure 2G). Finally, an M1 marker module score (16) revealed that monocytes derived from patients with severe calcified AVS harboring CH driver mutations showed polarization toward M1 macrophages, which are well established to be associated with progression to severe calcified AVS (13, 17, 18) (Figure 2, H and I, Supplemental Table 6). Taken together, these data show that monocytes from peripheral blood of patients with severe calcified AVS harboring *TET2* CH driver mutations exhibited a proinflammatory and procalcific gene expression signature.

### In vitro validation of the procalcific signature by mimicking CH in monocyte-derived macrophages.

**Figure 3:**
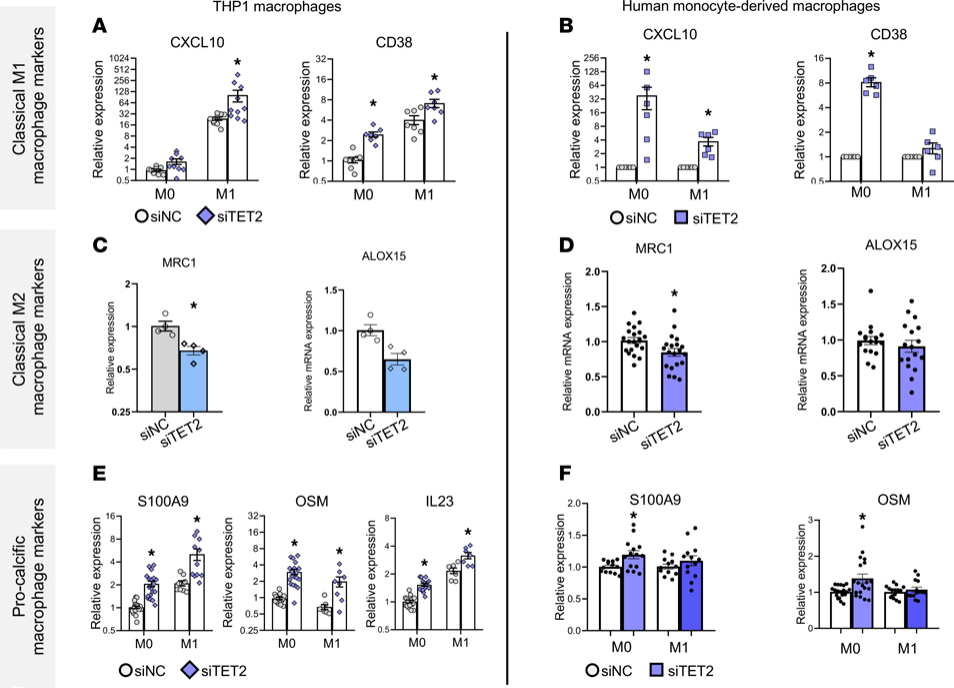
TET2 silencing enhances proinflammatory and procalcific gene expression in 2 macrophage models. (**A** and **B**) Expression of proinflammatory M1 markers in TET2-silenced M0 and M1 (**A**) THP1 macrophages (*n* = 7–10) and (**B**) human primary monocyte–derived macrophages (*n* = 6). (**C** and **D**) Expression of antiinflammatory M2 markers in TET2-silenced M0 (**C**) THP1 macrophages (*n* = 4) and (**D**) human primary monocyte–derived macrophages. (**E** and **F**) Expression of procalcific genes in TET2-silenced M0 and M1 (**E**) THP1 macrophages (*n* = 7–19) and (**F**) human primary monocyte–derived macrophages (*n* = 14–19). Statistical significance was assessed by 2-tailed Student’s *t* test within groups or, when comparing M0 and M1 groups, by 1-way ANOVA with Tukey’s multiple-comparison test. *P < 0.05. Data represent the mean ± SEM.

Since *TET2* CH driver mutations are loss-of-function mutations, we mimicked a CH phenotype by silencing *TET2* in THP1 macrophages and human primary monocyte–derived macrophages to address potential CH-mediated macrophage polarization in vitro. Silencing of *TET2* (Supplemental Figure 2A) resulted in increased expression of the classical M1 markers *CXCL10* and *CD38* in both resting (M0) and M1 macrophages (Figure 3, A and B), while expression the antiinflammatory (M2) markers *MRC1* and *ALOX15* was decreased in M0 and M2 macrophages (Figure 3, C and D) (19, 20). Moreover, concordant with the scRNA-Seq analyses of patient samples, CH gene silencing showed increased expression of the prototypical, procalcific secreted inflammatory factors *IL23*, *S100A9*, and *OSM* in both, M0 and M1 THP1 macrophages (Figure 3E) and upregulation of *S100A9* and *OSM* in both M0 and M1 human primary macrophages (Figure 3F).

**Figure 4:**
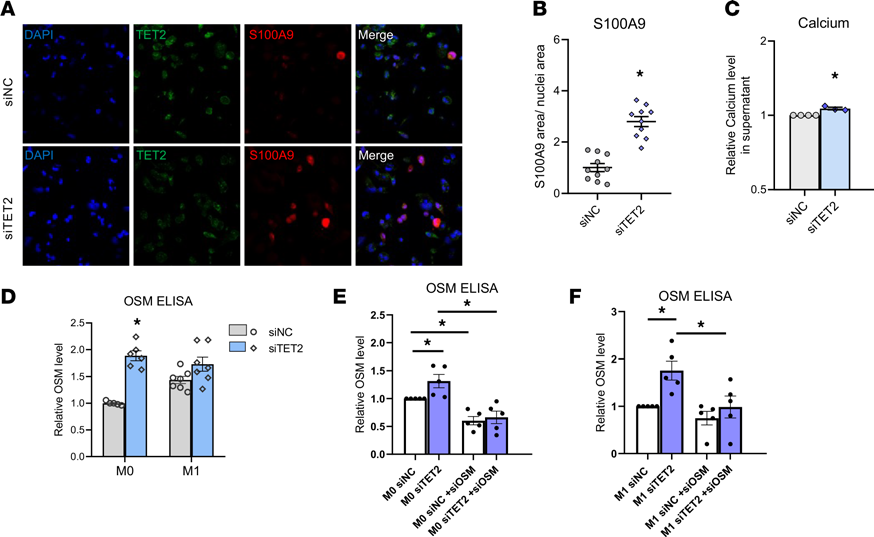
TET2 silencing promotes S100A9 accumulation and OSM-mediated calcium deposition. (**A**) Immunofluorescence analysis S100A9 (red) and TET2 (green) expression in control (top panels, siNC) and TET2-silenced (bottom panels, siTET2) macrophages. Original magnification, ×40. (**B**) Quantification of S100A9 detected by immunofluorescence in TET2-silenced macrophages (*n* = 10 areas, 2 independent experiments). (**C**) Calcium levels in the supernatant of TET2-silenced macrophages (*n* = 3). (**D**–**F**) ELISA of OSM protein secreted by TET2-silenced M0 and M1 THP1 macrophages (**D**) (*n* = 5–7), (**E**) M0 (*n* = 5), and (**F**) M1 (*n* = 5) siNC and siTET2 ± siOSM THP1 macrophages. Statistical significance was assessed by 2-tailed Student’s *t* test within groups or, when comparing M0 and M1 groups, by 1-way ANOVA with Tukey’s multiple-comparison test. *P < 0.05. Data represent the mean ± SEM.

Upregulation of S100A9, a calcium-binding protein and main component of procalcific extracellular vesicles (EVs) released by macrophages (15), was further validated in CH gene– silenced macrophages (Figure 4, A and B, and Supplemental Figure 2B). Co-occurrence of S100A9 and the extracellular vesicle marker CD9, along with increased calcium levels in the supernatant of CH gene–silenced THP1 macrophages (Figure 4C) suggests that the *TET2* CH driver gene influences S100A9-associated calcific EV secretion. Finally, secretion of OSM, an IL-6 family member known to mediate calcification in mesenchymal cells (21), was significantly increased in M0 and M1 CH gene–silenced THP1 and human primary macrophages (Figure 4, D–F).

Thus, transcript and protein data disclosed a markedly stimulated proinflammatory and procalcific potential in *TET2* gene–silenced macrophages.

### Paracrine effects of CH driver gene–silenced macrophages on the osteoblast-like potential of mesenchymal cells.

**Figure 5:**
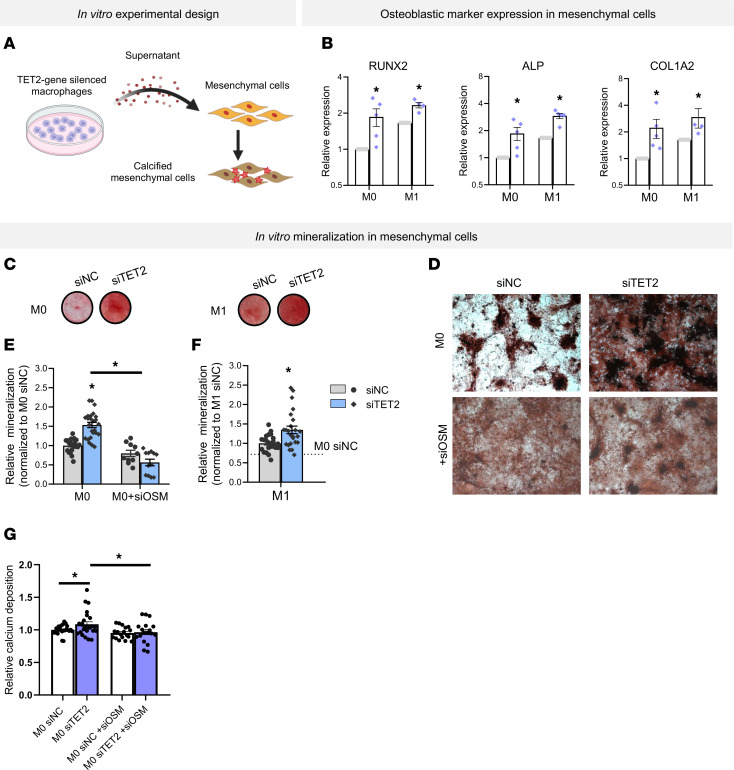
TET2 silencing stimulates mineralization of mesenchymal cells in vitro. (**A**) Schematic of the experimental design. (**B**) Expression of osteoblastic markers in mesenchymal cells indirectly cocultured with TET2 silenced or control M0 and M1 macrophages (*n* = 4–5; *n* = 4–5 independent experiments). (**C** and **D**) Representative mineralization of mesenchymal cells visualized by alizarin red S after indirect coculture with TET2 silenced M0 and M1 macrophages at (**C**) low and (**D**) high magnification. Original magnification, ×4. (**E**–**G**) Alizarin red S quantification of mineralization in mesenchymal cells after indirect coculture with (**E**) M0 TET2–silenced versus OSM- and TET2-silenced M0 THP1 macrophages (*n* = 10–25), (**F**) TET2- silenced M1 THP1 macrophages (*n* = 20–25), and (**G**) TET2-silenced versus OSM- and TET2-silenced M0 human primary macrophages (*n* = 18–24). Statistical significance was assessed by 2-tailed Student’s *t* test for within-group comparisons or by ordinary 1-way ANOVA with Tukey’s multiple-comparison test across groups. *P < 0.05. Data represent the mean ± SEM.

Macrophages can promote osteoblast conversion of mesenchymal cells but are not known to differentiate directly into osteoblasts, therefore, we hypothesized that CH-mutated macrophages may promote osteoblast-like differentiation by a secreted factor. To test this hypothesis, we cultured mesenchymal cells in the presence of conditioned medium derived from CH gene–silenced macrophages (Figure 5A). Mesenchymal cells exposed to the supernatant of M0 or M1 *TET2* gene–silenced macrophages displayed increased expression of the osteogenic markers *RUNX2, ALP*, and *COL1A2* (Figure 5B). In line with this observation, mesenchymal cells deposited increased calcified mineralized matrix after supernatant coculture with M0 and M1 *TET2* gene–silenced macrophages (Figure 5, C and D).

OSM is well established to promote osteoblast-like differentiation and ossifications of cells with mesenchymal origin in a paracrine manner. Given that GO term analyses of patient-derived monocytes identified OSM signaling in CH mutation carriers and, specifically, that silencing TET2 showed upregulation of OSM or OSM signaling (Figure 2, E and G, and Figure 3E), we addressed the potential contribution of OSM in CH gene–silenced macrophage-mediated mesenchymal cell calcification. Downregulation of OSM in TET2-silenced THP1 and human primary macrophages abrogated the augmentation of mesenchymal cell calcification induced by the supernatant of TET2-silenced macrophages (Figure 5, E–G). Thus, *TET2* CH gene silencing in macrophages also stimulated the osteoblast-like differentiation of mesenchymal cells in a paracrine manner, potentially via OSM (21–23).

### Mimicking TET2-mediated CH in mice enhances aortic valve calcification in vivo.

**Figure 6:**
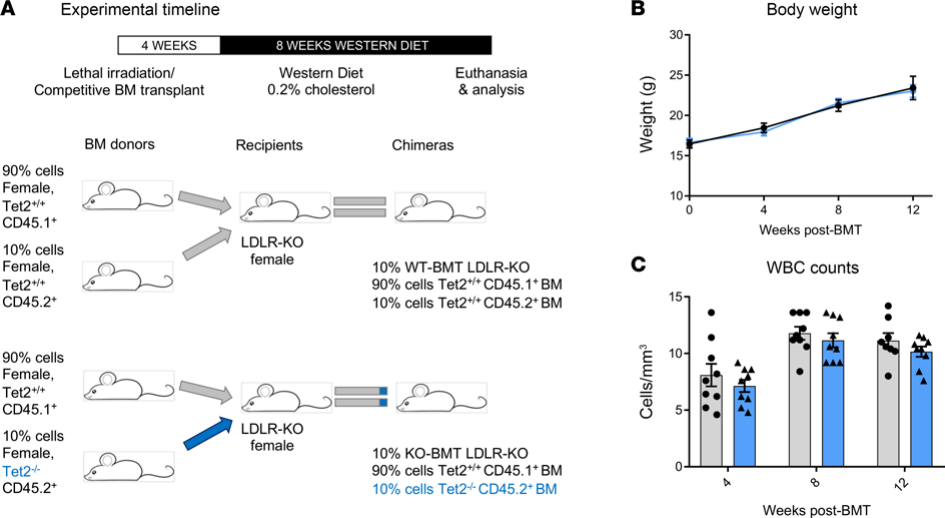
Experimental design and hematologic characterization of TET2 mutant BMT. (**A**) Workflow diagram for BMT studies along with (**B**) body weight (**C**) and WBC counts following BMT as shown by mutation status. Data represent the mean ± SEM. Statistical significance was assessed by 2-tailed Student’s *t* test between mutation types and only comparing data from the same time point.

**Figure 7:**
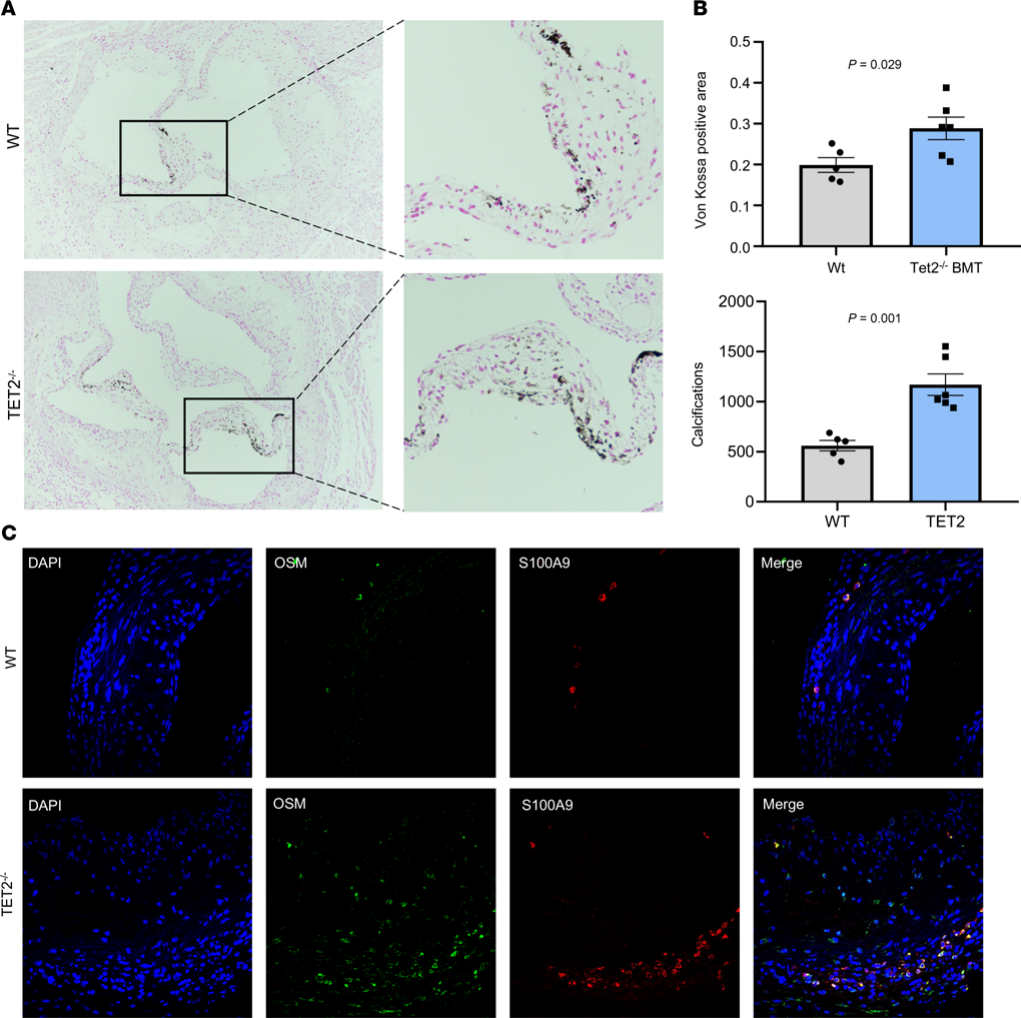
TET2-mutant BMT accelerates aortic valve calcification. (**A**) Von Kossa stainings of aortic valves in WT and *Tet2^−/−^* BMT mice with (**B**) quantification of positive calcified area (top) and number of calcific depositions (bottom) counted per image. (*n* = 5–6 animals/treatment; *n* = 5 images/animal). (**C**) Aortic valve staining with OSM and S100A9. Data represent the mean ± SEM. Statistical significance was assessed by 2-tailed Student’s *t* test between mutation types and only comparing data from the same time point. (**A**) and (**C**) Original magnification, ×4.

Finally, we assessed whether *TET2* CH driver mutations causally promote aortic valve calcification in vivo. Thus, we examined aortic valves of low-density lipoprotein receptor–deficient (*Ldlr^−/−^*) mice receiving 10% WT or *Tet2^–/–^* bone marrow transplants with a subsequent 8-week high-cholesterol diet (Figure 6, A–C, and Supplemental Figure 3, A–E). Valves from mice carrying *Tet2^−/−^* cells demonstrated an increase in the total calcified area (*P* = 0.029) along with increased numbers of calcification deposits, as evidenced by von Kossa staining (*P* = 0.001) (Figure 7, A and B). Moreover, in line with our cell culture model results, the aortic valve tissue of *Tet2^−/−^* recipient mice showed increased OSM and S100A9 levels (Figure 7C and Supplemental Figure 4, A and B), suggesting that the procalcific inflammatory signaling pathways identified in vitro wee also operative in vivo (24, 25). Consistently, transcriptomic analyses of *Tet2^−/−^* macrophages (Supplemental Figure 5) also revealed elevated *Osm* expression. Taken together, these data support the concept that *TET2* deficiency drives proinflammatory, procalcific signaling in vivo.

## Discussion

This is the first study, to our knowledge, demonstrating an association of CHIP with incident AVS, with an increased risk among patients with *TET2* and *ASXL1* mutations. Mechanistically, scRNA-Seq of peripheral blood cells revealed a procalcific gene signature in circulating monocytes derived from patients with severe calcified AVS harboring CH driver mutations in the *TET2* gene, one of the most prevalently mutated CH driver genes in patients with cardiovascular disease. In vitro studies mimicking CH document the procalcific potential of specifically *TET2* gene–silenced macrophages and identify OSM as a major driver of osteoblastic conversion of mesenchymal cells and cell mineralization in a paracrine fashion. Finally, a mouse model of *TET2* CH demonstrated increased calcification deposits and altered OSM levels in the aortic valve in vivo. Taken together, these data provide, to our knowledge, the first evidence that insults in the hematopoietic compartment alone may contribute to AVS and may help identify patients at high risk for progression to severe calcified disease. These advances may offer potential therapeutic avenues for this high-morbidity disease.

In the human studies reported here, the increased risk of AVS in the presence of CHIP across the All of Us, BioVU, and UK Biobanks is of a magnitude similar (~1.4) to the increased risk of coronary artery disease (CAD). The increased risk among non*–DNMT3A* mutation carriers also mimics previous work in CAD (26). The extent of AVS with CHIP is similar to that of AVS with diabetes and male sex, making CHIP among the strongest risk factors for AVS (27). Of note, previous work in institutional cohorts also found an increase in CHIP among patients with AVS (11) as well as an increase in progression of AVS among patients with CHIP (28). The current findings, both epidemiological and mechanistic, provide information on the pathophysiology underlying these prior data. Given the interest and difficulty in identifying biomarkers for AVS, CHIP may hold promise for risk stratification of patients with AVS even prior to diagnosis, allowing for earlier intervention, surveillance, or risk factor modification.

During the progression of aortic valve disease to severe calcified AVS, myofibroblasts in the valvular tissue transition to an osteoblast-like phenotype that promotes calcium phosphate deposition, culminating in valve dysfunction. Proinflammatory macrophages, recruited from circulating monocytes and observed in abundance in calcified human valve leaflets, are believed to promote the fibroblast-to-osteoblast differentiation and subsequent calcification in large part via paracrine mechanisms (17, 18, 29). The results of the present study show that CH is associated with circulating monocytes primed for activation of procalcific processes and signaling. *TET2* CH driver mutations appear to drive the expression of both pro-osteogenic genes as well as markers of proinflammatory M1 macrophage polarization in circulating monocytes obtained from patients harboring *TET2* CH driver mutations. Mimicking CH driver mutations in vitro by silencing *TET2* in macrophages recapitulated not only M1 macrophage polarization but also their procalcific potential on the transcript and protein levels. These results suggest that *TET2* CH driver mutations contributes to the development of severe calcified AVS by priming monocyte-derived macrophages recruited to the valvular tissue to precipitate mineralization and subsequent calcification. Such reasoning is also supported by our in vivo findings in mice, in which modeling *TET2* CH by bone marrow transplantation (BMT) of 10% *TET2*-mutant cells resulted in a significant increase in calcification deposits in the aortic valve tissue 12 weeks after BMT (*P* = 0.001).

Importantly, both, our scRNA-Seq analyses of circulating monocytes as well as our studies assessing potential paracrine mediators of enhanced osteoblast conversion of myofibroblasts and increased mineralization identified OSM as a potential paracrine mediator of *TET2* CH–associated valvular calcifications. OSM belongs to the IL-6 cytokine family and signals via the IL-6ST–gp130 receptor complex (21). In previous studies, IL-6 was identified and validated as a critical mediator of the pro-osteogenic calcification effects of M1 macrophage–derived conditioned media on valvular interstitial cells as well as a strong promoter of valve interstitial cell mineralization (30). Moreover, 2 recent GWAS identified a variant in the *IL6* gene promoter as one of the very few susceptibility genes for calcified AVS (31, 32). Thus, taken together, diseased aortic valves appeared to be sensitized to the OSM/IL-6 signaling pathway, and this sensitization was further amplified by the presence of TET2 CH driver mutations, which increased the expression and secretion of OSM in monocytes and monocyte-derived macrophages. However, future studies are necessary to demonstrate that blocking OSM signaling reduces mineralization deposits in aortic valve tissue in vivo in order to validate such a tailored therapeutic approach in patients harboring *TET2* CH driver mutations.

### Limitations.

We acknowledge several limitations of the current study. With respect to the human epidemiology, in the biobanking analysis, coding for aortic valve disease reflects an unstandardized diagnostic process. With regard to CH, there is an inherent inverse relationship between variant allele frequency (VAF) and the accuracy of somatic mutation calls. To account for this, we used a previously described approach that maintains maximum fidelity at low VAF (12). Given the chronic nature of both CH and AVS, future epidemiologic studies should also aim to clarify whether the presence of CH mutations affects the initiation of valvular calcification, the progression of existing disease, or both. With respect to our mechanistic studies, although we demonstrated that *TET2* CH driver gene–silenced macrophages released higher amounts of procalcific factors, which stimulate osteoblast-like differentiation of mesenchymal cells and deposition of calcified mineralized matrix in a paracrine fashion, we did not identify a receptor ligand in mesenchymal cells. Nevertheless, our in vivo studies in a mouse model mimicking *TET2* CH confirmed the results obtained in our cell culture model experiments with respect to increased mineralization associated with altered secretion of procalcific inflammatory factors in aortic valve tissue. We acknowledge that mice do not develop severe calcified AVS, so future studies should attempt to define the contribution of monocytes and macrophages with CH mutations to calcification within the human valve, perhaps when valves of patients with CH are explanted for surgical valve replacement.

Overall, our work highlights CH as a risk factor for AVS and identifies how perturbations in the blood can indirectly contribute to disease pathogenesis. This work also adds to the emerging understanding of how CH can contribute to disease through both direct invasion of CH-mutated cells into disease lesions as well as more recently recognized paracrine effects (6, 22, 33, 34).

## Methods

### Sex as a biological variable.

Both sexes were included in the human biobank and scRNA-Seq analyses, although sex was not analyzed as an independent biological variable. For in vivo aortic calcification analyses, our study exclusively examined female mice. Given that aortic valve calcification has been extensively characterized in male mice, the findings are expected to be relevant to both sexes.

### Study design and experimental procedures.

Human genetic and transcriptomics analyses were performed across 3 large biobanks (All of Us, BioVU, UK Biobank; *n* = 886,378) to assess associations between *TET2* mutations and AVS.

In vitro, macrophage *TET2* silencing and coculture assays with human aortic smooth muscle cells were used to assess procalcific signaling, OSM secretion, and mesenchymal mineralization.

Comprehensive experimental details, including sequencing protocols, cell culture methods, and statistical analyses, are provided in the Supplemental Methods.

### Statistics.

A full description of the statistical methods is provided in the Supplemental Methods. Briefly, group comparisons used *t* tests (2-tailed unless otherwise specified) or 2-way ANOVA with appropriate corrections; Cox regression was applied for biobank data; and transcriptomics analyses used FDR-adjusted thresholds. A *P* value of less than 0.05 was considered significant. Data are presented in the figures as mean ± SEM.

### Study approval.

The ethics review board of the Goethe University of Frankfurt (Frankfurt, Germany) approved the human study protocol, and the study complies with the Declaration of Helsinki. Animal experiments were approved by the institutional ethics committee of the Centro Nacional de Investigaciones Cardiovasculares (Madrid, Spain) and conducted in accordance with EU Directive 86/609/EEC. Full details are provided in the Supplemental Methods. Single-cell RNA-Seq of circulating monocytes from patients with degenerative AVS was conducted following ethics approval and informed consent. Murine BMT experiments in *Ldlr^−/−^* recipient mice were performed under approved institutional protocols to evaluate hematopoietic *TET2* loss and valve calcification.

### Data availability.

All raw and processed scRNA-Seq data have been deposited in the public archive ArrayExpress (https://www.ebi.ac.uk/biostudies/arrayexpress/studies/E-MTAB-15820). The Supporting Data Values file is provided for all graphs in the figures. Additional data and supportive information for this manuscript are available from the corresponding author upon request.

## Author contributions

WTA, MAR, BS, MAZ, NM, JJF, YP, CV, DS, SF, XY, AAPB, AW, MM, DJ, MS, MMR, and SG performed experiments and generated data. WTA, MAR, BS, SMP, MAZ, NM, JJF, YP, CV, AJS, EFE, YX, QSW, DS, SF, XY, AAPB, AW, MM, MVS, DJ, MS, MMR, and SG analyzed data and assisted with data interpretation. WTA, MAR, BS, SMP, MAZ, NM, JJF, YP, CV, AJS, EFE, YX, QSW, MVS, MVN, MRS, BRL, SD, AGB, and AMZ informed the study design. WTA, MAR, BS, SMP, MAZ, NM, JJF, YP, CV, AJS, EFE, YX, QSW, DS, SF, XY, AAPB, AW, MM, MVS, DJ, MS, MMR, MVN, SG, MRS, BRL, SD, AGB, and AMZ wrote the manuscript.

## Funding support

This work is the result of NIH funding, in part, and is subject to the NIH Public Access Policy. Through acceptance of this federal funding, the NIH has been given a right to make the work publicly available in PubMed Central.

- NIH grants F30HL147464 (to MAR), T32GM007347 (to MAR, AJS, and YP), F30DK127699 (to AJS), P30CA068485-19 (to MRS), and DP5OD029586 (to AGB).
- Leukemia and Lymphoma Society Clinical Scholar Award.
- E.P. Evans Foundation.
- Beverly and George Rawlings Directorship.
- Biff Rittenberg Foundation.
- Adventure Allie Fund (to MRS).
- Burroughs Wellcome Fund Career Award (to AGB).
- Dr Rolf M. Schwiete Foundation.
- German Research Foundation (SFB834/SFB1531, Projects B1 and S2, HERZBLUT Forschergruppe A02).
- European Research Council (Angiolnc) (to SD).
- CHIP-AVS (to AMZ).
- Neuroheart (to SD).
- German Center for Cardiovascular Research (DZHK).
- “la Caixa” Foundation, LCF/PR/HR22/52420011 (to JJF).
- This research was conducted using the UK Biobank resource, application 7089 and 43397.

## Supplementary Material

Supplemental data

Supporting data values

## Figures and Tables

**Table 1:**
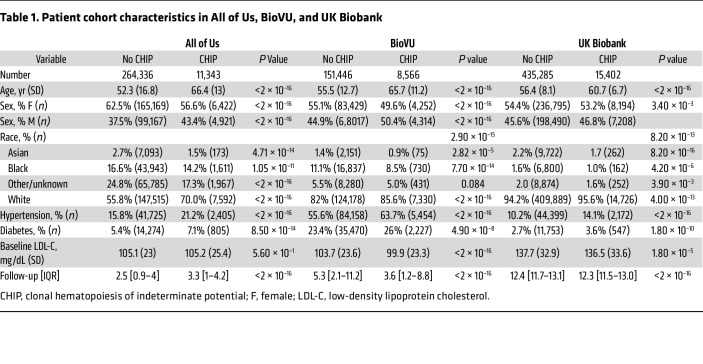
Patient cohort characteristics in All of Us, BioVU, and UK Biobank

## Version 1 11/18/2025

In-Press Preview

## Version 2 01/02/2026

Electronic publication
