# Supplementary material for: Clonal hematopoiesis activates procalcific pathways in macrophages and promotes aortic valve stenosis
Source: J Clin Invest. 2025 Nov 18;136(1):e171634. doi: 10.1172/JCI171634 (PMC12721883; doi:10.1172/JCI171634)
Supplement: Supplemental data [file jci-136-171634-s226.pdf]

Supplemental Table 1. CHIP mutation counts in the All Of Us, BioVU and UK Biobanks.

Gene	N
DNMT3A	9316
TET2	2041
ASXL1	1638
PPM1D	449
TP53	215
SRSF2	208
JAK2	128
SF3B1	109
GNB1	103
NF1	93
PRPF40B	87
GNAS	77
CBL	58
IDH2	48
ASXL2	44
CUX1	40
U2AF1	38
PRPF8	37
BRCC3	34
CREBBP	33
SETDB1	33
BCORL1	30
CEBPA	30
BCOR	26
PHIP	26
EP300	25
KRAS	23
STAG2	23

Gene	N
RAD21	22
ETV6	21
NRAS	20
SMC3	18
MPL	17
EZH2	16
PTPN11	16
KIT	15
KDM6A	14
PDS5B	14
PHF6	14
SUZ12	14
ZRSR2	13
WT1	12
BRAF	10
RUNX1	10
ETNK1	9
IDH1	8
CTCF	7
SETD2	7
CBLB	3
CSF3R	3
GATA2	3
IKZF1	3
SMC1A	2
EED	1
SETBP1	1
U2AF2	1

Supplemental Table 2. ICD codes for aortic valve disease, myeloid neoplasms and valve replacement. A combination of ICD9 and ICD10 codes was used. ICD = international classification of diseases.

Criterion	Codes
**Aortic Valve Disease ICD9 Codes**	
Mitral valve stenosis and aortic valve stenosis	396.0
Mitral valve insufficiency and aortic valve stenosis	396.2
Aortic valve disorders	424.1
**Aortic Valve Disease ICD10 Codes**	
Nonrheumatic aortic (valve) stenosis	I35.0
Nonrheumatic aortic (valve) stenosis with insufficiency	I35.2
**Myeloid Neoplasm ICD9 Codes**	
Myeloid leukemia	205*
Polycythemia vera	238.4
Neoplasm of uncertain behavior of other lymphatic and hematopoietic tissues (includes myelodysplastic syndromes)	238.7*
**Myeloid Neoplasm ICD10 Codes**	
Myeloid leukemia	C92*
Polycythemia vera	D45
Myelodysplastic syndromes	D46*
Chronic myeloproliferative disease	D47.1
Essential thrombocythemia	D47.3
**Heart Transplant and Valve Replacement ICD9 Codes**	
Heart replaced by transplant	V42.1
Heart valve replaced by transplant	V42.2*
Heart replaced by other means	V43.2
Heart valve replaced by other means	V43.3
**Heart Transplant and Valve Replacement ICD10 Codes**	
History of heart transplant	Z94.1
History of heart and lung transplant	Z94.3
Presence of prosthetic heart valve	Z95.2
Presence of xenogenic heart valve	Z95.3
Presence of other heart-valve replacement	Z95.4
Presence of other cardiac implants and grafts	Z95.81*

Supplemental Table 3. Demographic characteristics of AS patients with and without CHIP mutations included in scRNA-seq study.

Sample	Age	eGFR	Cereb Vasc Dis	Periph Art Occ Dis	COPD	Diabetes	Hypertension	Prev_MI	Prev_Stroke	Prev_TIA	Prev_PCI	CAD	Afib
No_CH_1	87	46	0	0	0	0	1	0	0	0	1	3	0
No_CH_2	84	62	0	0	0	0	1	0	0	0	0	0	1
No_CH_3	83	71.4	0	0	0	0	1	0	0	0	0	0	1
TET2_1	88	32.9	0	0	0	1	1	0	0	0	1	1	0
TET2_2	86	40.9	0	0	0	0	1	0	1	0	0	0	1
TET2_3	91	75.4	0	0	0	0	1	0	0	0	1	2	1
TET2_4	77	59.9	0	0	0	0	1	0	1	0	0	0	1
TET2_5	80	68.2	0	0	0	0	1	0	0	0	0	0	1

Sample	EF	P_mean	Uric acid	NTproBNP	Trop	CK	CRP	IL6	Hb	HKT	Thr	Leukocytes
No_CH_1	55	45	47	1317	21	65	0.1	14	10.1	31.7	254	7.72
No_CH_2	45	53	49	1933	21	56	0.14	3.2	12.9	37.9	297	7.41
No_CH_3	65	74	39	2115	16	47	0.44	4	12.6	37.4	169	6.61
TET2_1	50	70	61	2077	38	124	0.08	3	13.3	40	158	7.03
TET2_2	65	40	45	5714	19	68	0.11	13.8	12.3	37.2	163	8.07
TET2_3	40	28	27	3519	278	90	0.72	9.6	10	28.7	134	3.39
TET2_4	65	71	36	1686	7	96	0.29	8.8	14.4	41.3	226	6.22
TET2_5	60	41	31	1119	20	120	0.06	2	11.2	31.5	197	4.95

Supplemental Table 4. Non-synonymous sequence variations of CH patients detected by targeted genome sequencing

Sample	Hugo Symbol	Chromosome	Positon (hg19)	Exon	DNA Change	Protein Change	DOMAIN	VAF (%)
TET2_1	*TET2*	4	106164074-106164075	5	c.3584_3585insA	p.A1196Cfs*2	CD	20
TET2_2	*TET2*	4	106197302	11	c.5635G>A	p.E1879K	DD	2,63
TET2_3	*TET2*	4	106190842	9	c.4120T>C	p.C1374R	DD	26,7
TET2_4	*TET2*	4	106155582	3	c.483dup	p.D162Rfs*9	CD	37,4
TET2_5	*TET2*	4	106157229-106157230	3	c.2108_2130dup	p.E711*	CD	2,76

Supplemental Table 5. Single cell RNA-sequencing metrics

No CH	**No CH 1**	**No CH 2**	**No CH 3**
Number of Reads	1028059260	1019510963	1011465852
Reads With Valid Barcodes	0.976	0.977	0.976
Sequencing Saturation	0.818	0.867	0.811
Q30 Bases in RNA read	0.910	0.909	0.909
Reads Mapped to Genome: Unique	0.908	0.904	0.919
Fraction of Reads in Cells	0.880	0.891	0.872
Mean UMI per Cell	7039	8532	10606
Mean Genes per Cell	1815	2081	2533
Total Genes Detected	21786	21317	21855
Total Cells	11046	6678	8011

TET2	**TET2_1**	**TET2_2**	**TET2_3**	**TET2_4**	**TET2_5**
Number of Reads	1037102181	1128373357	1128615294	1041567557	1119936989
Reads With Valid Barcodes	0.976	0.976	0.976	0.977	0.977
Sequencing Saturation	0.816	0.828	0.840	0.820	0.817
Q30 Bases in RNA read	0.899	0.901	0.910	0.898	0.900
Reads Mapped to Genome: Unique	0.912	0.911	0.907	0.909	0.915
Fraction of Reads in Cells	0.907	0.903	0.924	0.909	0.871
Mean UMI per Cell	10257	8889	8615	8127	8193
Mean Genes per Cell	2505	2091	2144	2062	2060
Total Genes Detected	22380	22330	21959	22103	22427
Total Cells	8408	9524	9199	10084	11126

Supplemental Table 6. M1 and osteogenic markers for module scores

**Module Score**	**Markers**
CXCL10	M1
IL1B	M1
IL2	M1
IL6	M1
IL6ST	M1
CXCL8	M1
TNF	M1
IFNG	M1
NFKB1	M1
OSM	Osteogenic
S100A9	Osteogenic
IL23A	Osteogenic
RUNX2	Osteogenic
ALPL	Osteogenic
SPP1	Osteogenic
BMP2	Osteogenic
BMP4	Osteogenic

Supplemental Table 7. Oligonucleotides used in this study.

Primer	Application	Sequence 5'→3'
DNMT3A For	qPCR	TATTGATGAGCGCACAAGAGAGC
DNMT3A Rev	qPCR	GGGTGTTCCAGGGTAACATTGAG
TET2 For	qPCR	AAGCAAGATCCCAAGGAAGT
TET2 Rev	qPCR	GCAAATGAGACTCCAGTTT
RPLP0 For	qPCR	ATCCGTCTCCACAGACAAGG
RPLP0 Rev	qPCR	TCGACAATGGCAGCATCTAC
CXCL10 For	qPCR	GCTTCCAAGGATGGACCACA
CXCL10 Rev	qPCR	GCAGGGTCAGAACATCCACT
CD38 For	qPCR	CTGCCTGTGATGTGGTCCAT
CD38 Rev	qPCR	ATCACCCAGGCCTCTAGTGT
CCL22 For	qPCR	ATTACGTCCGTTACCGTCTG
CCL22 Rev	qPCR	TAGGCTCTTCATTGGCTCAG
ALOX15 For	qPCR	TTCTATGCCCAAGATGCGCT
ALOX15 Rev	qPCR	TGCAGCCCGATTTCAGTGAT
IL-23 For	qPCR	GAGCCTTCTCTGCTCCCTGATA
IL-23 Rev	qPCR	GACTGAGGCTTGGAATCTGCTG
OSM For	qPCR	TACTGCTCACACAGAGGACG
OSM Rev	qPCR	CTGCTCTAAGTCGGCCAGTC
S100A9 For	qPCR	GACTTGCAAAATGTCGCAGC
S100A9 Rev	qPCR	GCCCCAGCTTCACAGAGTAT
RUNX2 For	qPCR	CGGAATGCCTCTGCTGTTATG
RUNX2 Rev	qPCR	GGGAGGATTTGTGAAGACGGT
ALP For	qPCR	CTATCCTGGCTCCGTGCTCC
ALP Rev	qPCR	AGAGATGCAATCGACGTGGG
COL1A2 For	qPCR	GGATGAGGAGACTGGCAACC
COL1A2 Rev	qPCR	TGCCCTCAGCAACAAGTTCA

Supplemental Table 8. Antibodies used in this study

**Antibody**	**Host species, clonality**	**Application, dilution**	**Vendor**	**Catalog#**
Human anti-TET2 (D6B9Y)	Rabbit, monoclonal	WB 1:2000, IF 1:50	Cell Signaling	18950
Human anti-GAPDH (14C10)	Rabbit, monoclonal	WB 1:5000	Cell Signaling	2118
Anti-rabbit Ig HRP-conjugated	Goat, polyclonal	WB 1:2000	Dako	P0448
Human anti-S100A9	Mouse, monoclonal	IF 1:50	Origene	UM870066
Human anti-CD9 (E8L5J)	Rabbit, monoclonal	IF 1:50	Cell Signaling	98327S
Human/mouse Anti-Oncostatin M	Rabbit, polyclonal	IHC 1:50	Invitrogen	PA5-76861
Mouse anti-S100A9	Goat, polyclonal	IHC 1:50	Invitrogen	A31573
Anti-rabbit Alexa Fluor 488	Donkey, polyclonal	IF 1:200	Invitrogen	A-21206
Anti-mouse Alexa Fluor 647	Donkey, polyclonal	IF 1:200	Invitrogen	A-31571

Supplemental Figure 1

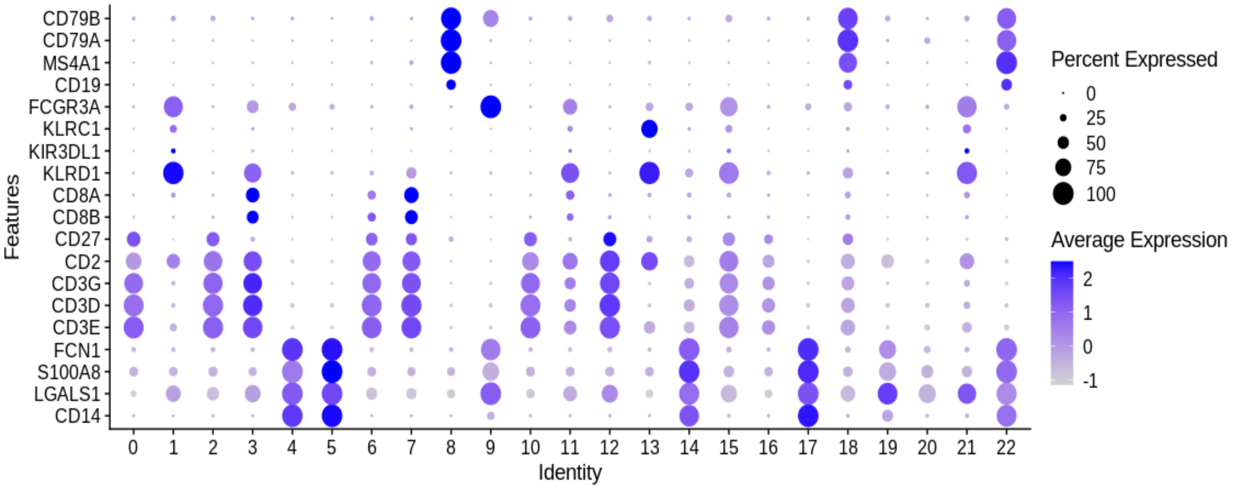

Supplemental Figure 2

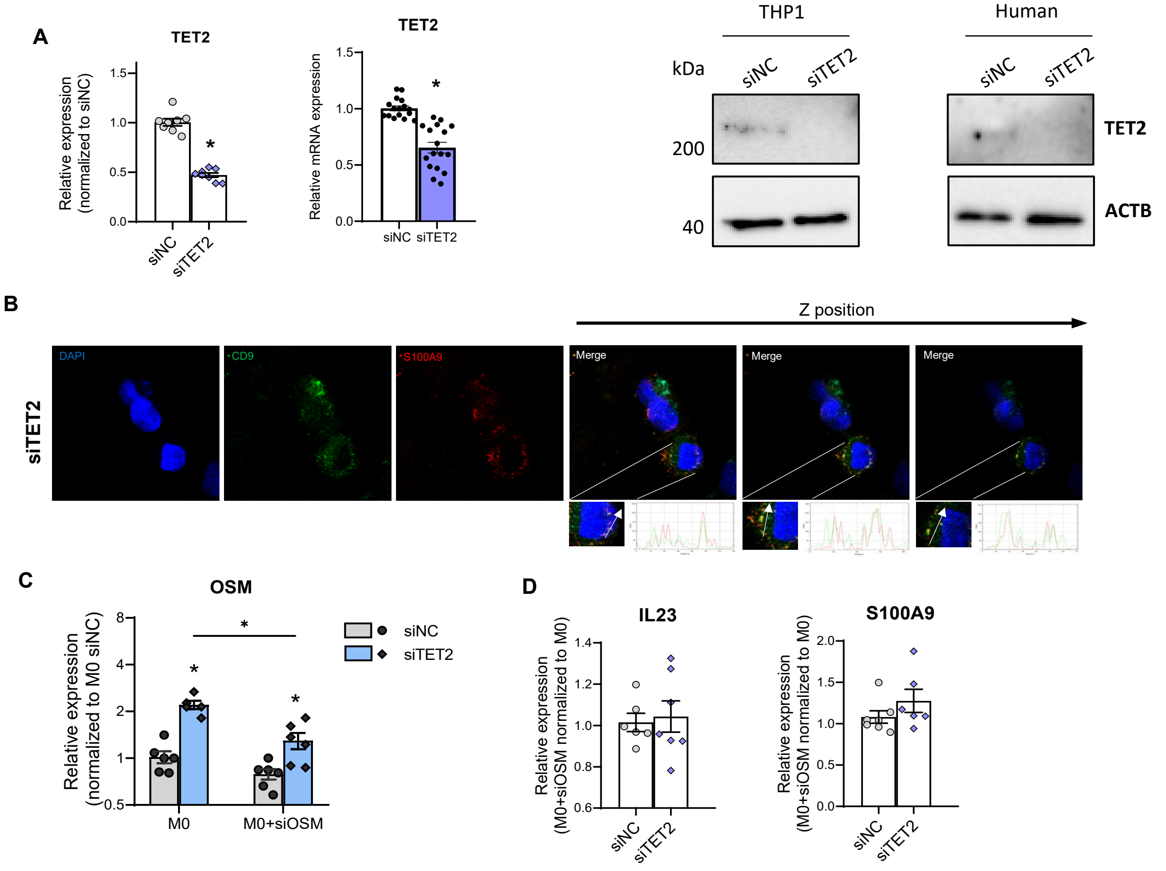

Supplemental Figure 3

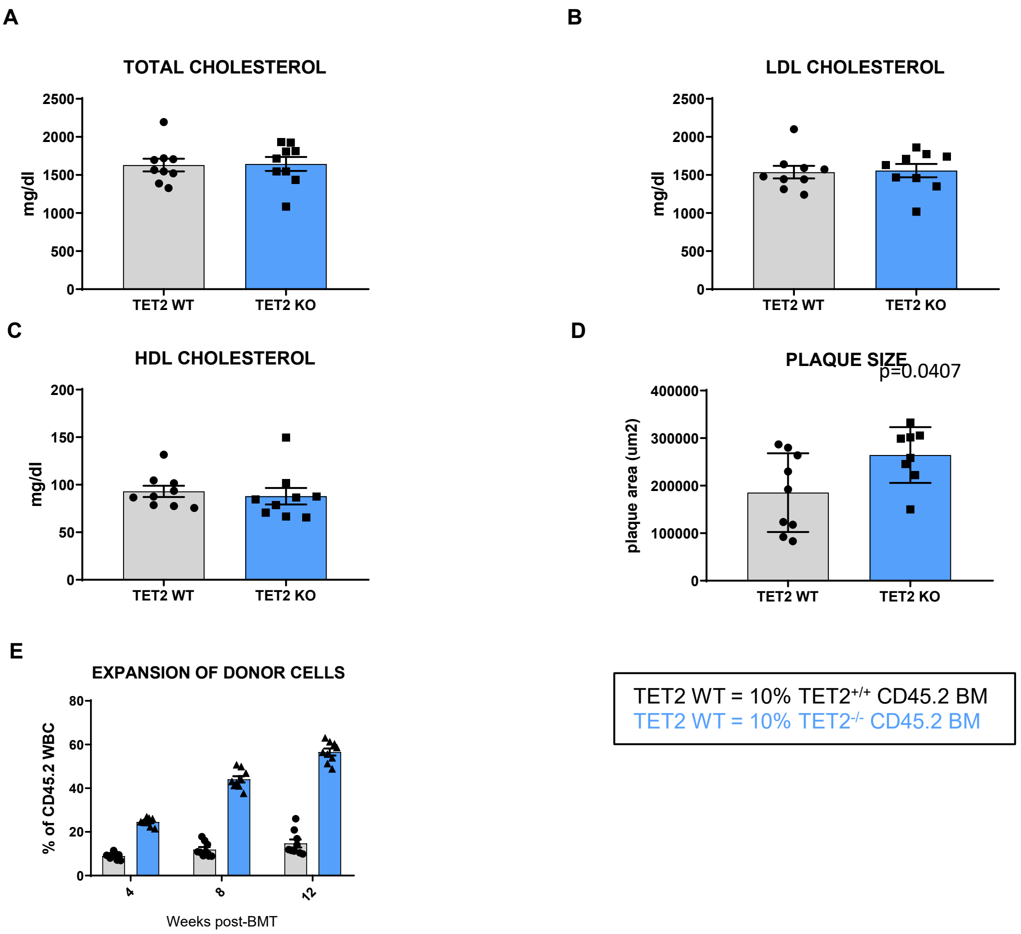

Supplemental Figure 4

***WT* 40x magnification**

A

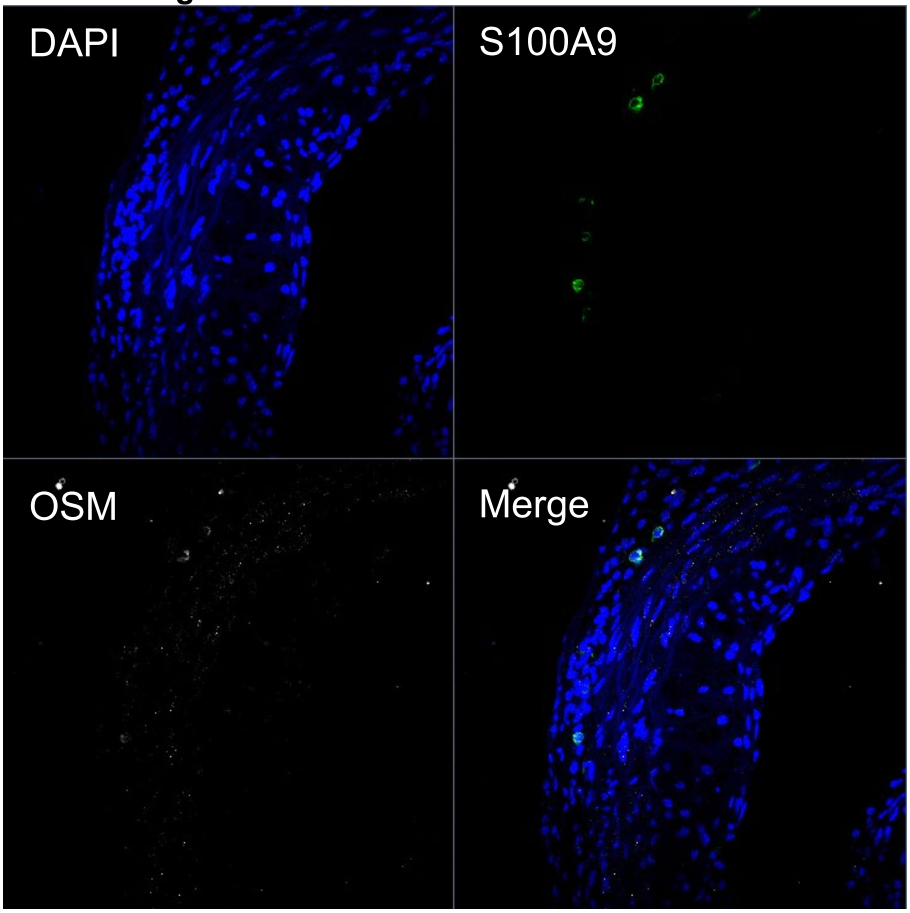

***Tet2-/-* 40x magnification**

B

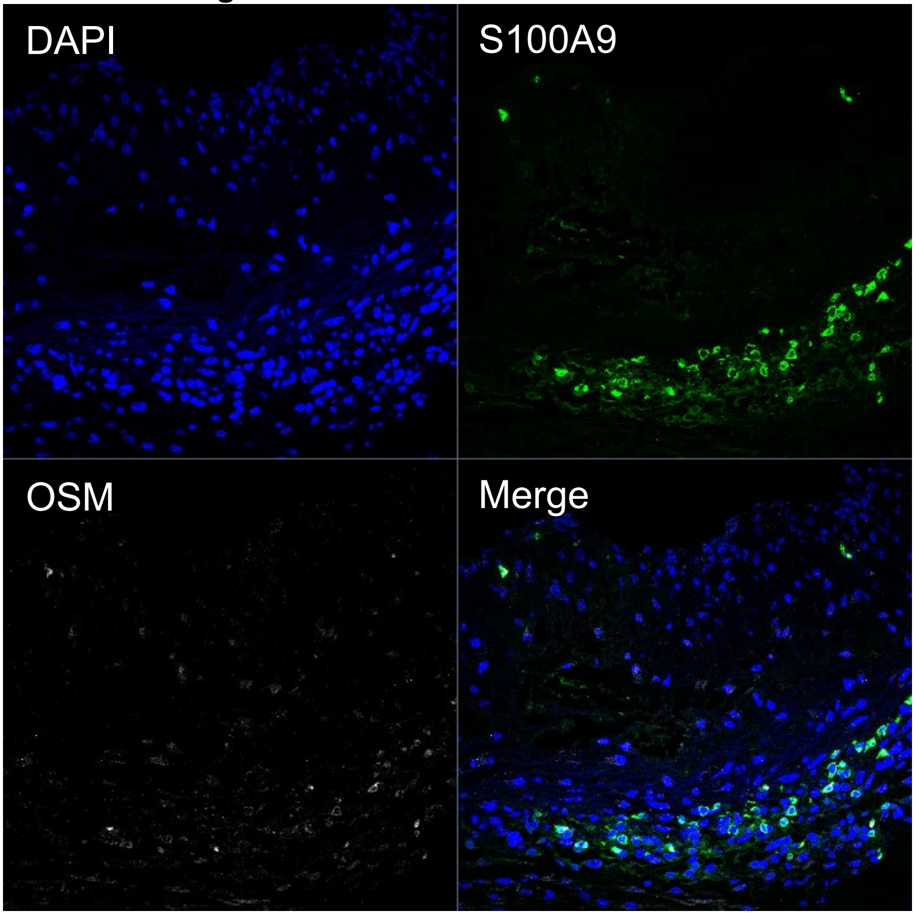

Supplemental Figure 5.

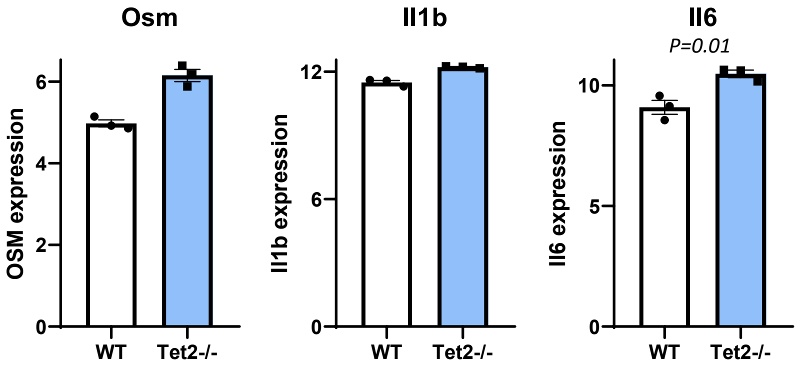

Online Methods

Sex as a biological variable

Both sexes were included in the human biobank and scRNA-seq analyses, although sex was not analyzed as an independent biological variable. For in vivo aortic calcification analyses, our study exclusively examined female mice. Given that aortic valve calcification has been extensively characterized in male mice, the findings are expected to be relevant to both sexes.

Biobank Populations

The meta-analysis of the UK Biobank, All Of Us, and BioVU included individual-level data from 886,378 participants across the three biobanks with sequencing data. CHIP was detected in these individuals using previously described methods.(1) AVS diagnosis and covariates were described using ICD codes (**Supplemental Table 2**). (2–4)

Patient recruitment for scRNA-seq in Frankfurt

We analyzed single-cell sequencing signatures of gene expression in circulating monocytes obtained from patients with severe degenerative aortic valve stenosis. Patients with evidence for acute inflammatory or hematological disease were excluded, and all patients were recruited prior to the outbreak of COVID-19 in Europe (August 2019, and November 2019). Patients were enrolled 5-26 months after undergoing TAVI procedure. All patients provided written informed consent for the study. The ethics review board of the Goethe University of Frankfurt, Germany, approved the protocol, and the study complies with the Declaration of Helsinki.

Targeted genomic sequencing for Frankfurt cohort screened for scRNA-seq

Next-generation sequencing was commercially performed by MLLDxGmbH, München, Germany. In brief, DNA was isolated with the MagNaPure System (Roche Diagnostics, Mannheim, Germany) from mononuclear cells after lysis of erythrocytes. The patients' libraries were generated with the Nextera Flex for enrichment kit (Illumina, San Diego, CA, USA) and sequences for DNMT3A and TET2 enriched with the IDT xGen hybridization capture of DNA libraries protocol and customized probes (IDT, Coralville, IA, USA). The libraries were sequenced on an Illumina NovaSeq 6000 with a mean coverage of 2147× and a minimum coverage of 400×, reaching a sensitivity of 2%. Reads were mapped to the reference genome (UCSC hg19) using Isaac aligner (v2.10.12) and a small somatic variant calling was performed with Pisces (v5.1.3.60). Protein truncating variants were classified as mutation. Non-synonymous changes were included, if they were well annotated (several definite submissions to COSMIC, IRAC, or ClinVAR). Other non-protein truncating variants were defined as variants of uncertain significance.

Single-cell RNA-sequencing

For single-cell RNA sequencing, blood was obtained from patients and centrifuged on a Ficoll gradient, and mononuclear cells were used for droplet single-cell RNA sequencing with Chromium Controller and Chromium Next GEM Single Cell 3' GEM, Library and Gel Bead Kit version 3.1 reagents [10X Genomics], as previously described(5). Libraries were sequenced using paired-end sequencing by GenomeScan (Leiden, Netherlands). Single-cell expression data were processed using the Cell Ranger Single Cell Software Suite (10X Genomics) to perform quality control, sample demultiplexing, barcode processing, and single-cell 3′ gene counting and were then aligned to the human reference genome GRCh38.

Data integration was performed by Seurat version 4 (Satija Lab)(6) and statistical analysis of differential expression of genes was performed with the FindMarkers function in the Seurat

package with adjusted p-values less than 0.05 considered significant. AddModuleScore function was utilized to gain insights into gene programs with genes shown in **Supplemental Table 6**. This function calculates the average expression levels of each described program (cluster) on single cell level, subtracted by the aggregated expression of control feature sets. All analyzed features are binned based on averaged expression, and the control features are randomly selected from each bin. Gene ontology terms were generated using the functional annotation tool Metascape (Metascape Team)(7).

Cell culture and transfection

THP1 cells were obtained from the German Collection of Microorganisms and Cell Cultures (DSMZ, #ACC16) and maintained in RPMI 1640 media supplemented with 2 mM L-Glutamine, 50 U/mL Penicillin and Streptomycin, 10% heat-inactivated FBS and 10 mM HEPES at 5% CO_2 and 37°C.

Differentiation of THP1 monocytes into THP1-derived macrophages was induced by exposure of 5×10^5 THP1 monocytes/6-well to 100 ng/mL Phorbol 12-myristate 13-acetate (PMA, Sigma Aldrich) for 48 h followed by 24 h of culture in media without PMA.

Peripheral blood mononuclear cells (PBMCs) were isolated from healthy donor buffy coats by Ficoll density gradient centrifugation. Monocytes were enriched by CD14+ magnetic bead separation and cultured in RPMI 1640 supplemented with 10% FBS, penicillin/streptomycin, and recombinant human GM-CSF (50 ng/mL) for 7 days. Media and cytokines were replenished every 2–3 days. At day 7, adherent cells displayed macrophage morphology and were used for downstream experiments.

Genes of interest were downregulated in THP1 macrophages by RNA interference with 50 nM siRNA and siTran2.0 transfection reagent (Origene) following the manufacturer's instructions. The media was refreshed 18 h post transfection. TET2 (HSS123253) was silenced with Stealth

RNAi siRNAs (Invitrogen) along with a negative control of median GC content (Invitrogen, #12935300). The siRNA against OSM was of ON-TARGETplus type (Dharmacon, #L-010528-00-0005).

Human aortic smooth muscle cells (hASMCS) were used as cells of mesenchymal origin. They were purchased from PromoCell (#C12533, Lot 424Z011.4) and kept in DMEM supplemented with 2 mM L-Glutamine, 50 U/mL Penicillin and Streptomycin, 10% heat-inactivated FBS at 5% CO_2 and 37°C. ASMCs were seeded in 12-well plates for RNA extraction and qPCR analysis or in 48-well plates for detection of mineralization at a density of 10.000 cells/cm^2. Osteoblastic-like differentiation of hASMCs, which had adhered in growth media overnight, was induced by addition of 10 mM β-glycerol phosphate, 100 nM dexamethasone and 50 µM L-ascorbic acid.

Macrophage polarization and indirect co-culture

THP1 cells were polarized into pro-inflammatory M1 or anti-inflammatory M2 macrophages 36 h post transfection with 20 ng/mL IFN-γ and 10 pg/mL LPS or 25 ng/mL IL-4 and 25 ng/mL IL-13, respectively, for 48 h. All cytokines were purchased from PeproTech. Human primary monocytes were differentiated into macrophages from isolated PBMCs with CD14+ sorting and then cultured and adhered to plates in RPMI 1640 with 10% FBS and 20 ng/mL GM-CSF for 7 days (media refreshed every ~3 days).

For indirect co-culture experiments, the cell culture supernatant produced by macrophages for three days was pooled and concentrated to 500 µl using Ultra-15 centrifugal filter units (Amicon, MWCO 3 kDa). The volume of the supernatant was adjusted with mesenchymal cell media to obtain a three-fold concentrate added to mesenchymal cells with an equal volume of target cell media. The mesenchymal cell media mixed with macrophage supernatant was

refreshed every three to four days. Gene expression and mineralization of mesenchymal cells were assessed after 10 d and 21 d of indirect co-culture with macrophages, respectively.

RNA extraction and quantitative real-time PCR

Total RNA was extracted using the RNeasy Mini Kit (Qiagen) according to the manufacturer's protocol. RNA quantity and quality were determined by UV-Vis-spectrophotometry (NanoDrop2000, Thermo scientific) followed by reverse transcription of 500-1000 ng RNA with M-MLV Reverse Transcriptase (Invitrogen) as recommended by the manufacturer.

For quantitative real-time PCR (qPCR) analysis, intron-spanning primers were designed using the Primer BLAST tool.[4] Primers were confirmed to amplify a specific product meeting qPCR efficiency of 100+/-15%. All qPCR primers are listed in **Supplemental Table 7**. SYBR Green-based assays were performed in a 10 µl reaction consisting of 5 µl Fast SYBR Green Master Mix 2X (Applied Biosystems), 2 µl nuclease-free H_20, 0.25 µl of each 10 mM forward and reverse primer and 10 ng cDNA template in 2.5 µl. Assays were carried out in 384-well plates in technical triplicates and run on a ViiA 7 instrument with Quant Studio Real-Time PCR Software (both Applied Biosystems). The thermal profile consisted of 95°C for 20 sec followed by 40 cycles of 95°C for 1 sec and 60°C for 20 sec and a final melt curve stage (95°C 15 sec, 60°C 1 min and 95°C 15 sec). Data was analyzed using the ΔΔCt method and RPLP0 as reference gene. Data is represented as relative mRNA level ($2^{-\Delta\Delta Ct}$) normalized to the expression level in the respective control.

Indirect immunofluorescence

For immunofluorescence analysis, cells were grown on 18 mm round glass coverslips in 12-well plates and washed with PBS before fixing them with 4% paraformaldehyde at room temperature for 15 min. After washing the cells three times with PBS for 5 min, they were

permeabilized with 0,1% Triton X-100 in PBS for 5 min and washed three times with PBS for 5 min. Cells were blocked in 5% donkey serum in PBS for 45 min followed by incubation in primary antibody, diluted in 5% donkey serum in PBS, in a humid chamber for 1 h (**Supplemental Table 8**). After three washing steps with PBS for 5 min, incubation in fluorescently labeled secondary antibody and 1 µg/mL DAPI was performed in a humid chamber in the dark for 1 h. Finally, the cells were washed three times with PBS for 5 min, air-dried and mounted on microscope slides. Images were acquired using the Leica SP8 Confocal Microscope. For quantification, five areas per experiment, each comprising 30-40 cells, were randomly chosen to determine the positive pixel area of a target normalized to the respective nuclei area stained with DAPI. Images were analyzed using LAS X and Image J software.[5]

Mice

Animal experiments followed protocols approved by the Institutional Ethics Committee at the Centro Nacional de Investigaciones Cardiovasculares and conformed to EU Directive 86/609/EEC and Recommendation 2007/526/EC regarding the protection of animals used for experimental and other scientific purposes, enforced in Spanish law under Real Decreto 1201/2005. *Ldlr-/-* mice carrying the CD45.1 isoform of the CD45 hematopoietic antigen were generated by crossing *Ldlr-/-* mice from Jackson Laboratories and B6.SJL-PtprcaPepcb/BoyCrl mice from Charles River Laboratories. *Tet2-/-* mice were obtained from Jackson Laboratories. All mice were maintained on a 12-h light/dark schedule in a specific pathogen-free animal facility in individually ventilated cages and given food and water ad libitum.

Competitive bone marrow transplantation and atherosclerosis induction

CD45.1+ *Ldlr-/-* recipients were transplanted with suspensions of BM cells containing 10% CD45.2+ *Tet2-/-* cells and 90% CD45.1+ *Tet2+/+* cells (10% KO-BMT mice) or 10 % CD45.2+ *Tet2+/+* cells and 90% CD45.1+ *Tet2+/+* cells (10% WT-BMT mice). Recipient *Ldlr-/-* mice were exposed to two doses of 500 rad three hours apart. After the second irradiation, each recipient mouse was injected a total of 10 million bone marrow cells intravenously. Recipient mice were provided with antibiotics in the drinking water for 3 weeks. Starting 4 weeks after BMT, transplanted mice consumed a high fat high cholesterol western diet (0.2% cholesterol, ENVIGO TD88137) for 8 weeks. After 8 weeks on this diet, mice were euthanized and their aortic roots were collected and fixed with 4% paraformaldehyde in PBS overnight at 4°C. Samples were then dehydrated and embedded in paraffin for sectioning. Histological sections comprising the aortic root were cut at a thickness of 4 μm, and stained with hematoxylin and eosin for plaque size quantification. Plaque size in the aortic root sections was quantified by computer-assisted morphometric analysis of microscopy images.

Cholesterol was measured in serum collected before euthanasia with an enzymatic assay (Cholesterol E, WAKO Diagnostics), following manufacturer instructions.

For flow cytometry and hematological analysis, peripheral blood was collected from the facial vein into EDTA-coated tubes. White blood cell counts were determined with a hematological counter. For flow cytometry analysis, white blood cells were stained with antibodies against CD45.1 and CD45.2 to determine the percentage of donor cells. A BD FACSymphony Cytometer (BD Bioscience) was used for data acquisition, and FlowJo Software Data for analysis.

Von Kossa staining and immunohistochemistry

An operator who was blinded to genotype performed von Kossa staining following standard procedure for Silver plating kit according to von Kossa (Merck, cat# 1003620001). In principle,

silver nitrate solution reacts with carbonate and phosphate ions of the calcium in the stored calcium deposits and displace the calcium ions. These silver ions are reduced to metallic silver by exposure to strong light and this silver is evaluated by microscopy. Slides were deparaffinized in conventional manner and rehydrated in descending alcohol series.

After dehydration and clearing with xylene (paraffin was melted for 30 min at 70°C, with 2x10 min. in xylene, 5 min. 100% in ethanol, 5 min. 95% in ethanol, 5 min. 80% in ethanol, 5 min. 70 % in ethanol, 5 min. 50 % in ethanol, 5 min. in distilled water), histological samples were stained and counterstained (nuclear fast red/Kernechtrot), per kit recommendations, and mounted and stored until analysis. Microscopic quantification of the sum calcification of the aortic valve was under taken at 10x to capture the entire valve/aortic root area. Images were taken under a Nikon Eclipse Ci microscope. Operator was blinded to the treatment of the animals.

Von Kossa staining analysis was performed with ImageJ to ascertain relative calcium deposition area and number of calcium deposits. A macro script was utilized to analyze images to remove operator bias. Briefly, images were processed in batch by being converted to 32-bit images, with upper limit thresholds decreased to 152 (lower limit was not adjusted), and masking performed to reveal the distinct, dark von Kossa stain and remove lighter background. The functions "measure" and "analyze particles" were used to characterize the valves and provide quantified assessment of readouts. Percent area and number of counted depositions/particles are reported. Five images of the aortic valve area were utilized per animal.

Staining of paraffin sections with OSM and S1009 involved standard workflows for dehydration and clearing with xylene as described above. Following clearing, antigen retrieval was performed in 0.01 M citrate buffer pH=6,0 for 90 seconds in under pressure, with 4°C water cooling afterwards followed by washing 3x5 min. in PBS/0,1% Triton, blocking with 3% BSA, 20mM MgCl2, 0,3% Triton, 5% Donkey serum/PBS (Histoblock) for 60 min at room

temperature. Anti-Oncostatin M (Invitrogen #PA5-76861) and anti-S100A9 (R&D #AF2065) were both diluted 1:50 in Histoblock solution and incubate 4°C overnight. Slides were washed 3x5 min. PBS/0,1% Triton, incubated with donkey anti-rabbit 647 (Invitrogen # A31573) and donkey anti-goat 488 (Invitrogen #A11055) in PBS/0,1% Triton solution for 60 min. at room temperature in the dark, then washed 3x5 min. PBS/0,1% Triton and mounted with medium with Höchst 33342 and store at 4°C in the dark. Images were taken with LSM 780.

OSM ELISA

The amount of OSM secreted from THP1 macrophages was quantified with a calorimetric human OSM ELISA (Abcam, #ab215543). The cell culture supernatant was diluted 1:2 with assay buffer and processed as described by the manufacturer. After addition of stop solution, the plate was read at 450 nm with a microplate reader (Biotek, Synergy HT).

Calcium Assay

Calcium levels of cell culture supernatants were determined using a Calcium Calorimetric Assay Kit (Sigma, #MAK022) according to the manufacturer's instruction. In brief, 50 µl supernatant diluted 1:5 was mixed with 90 µl Chromogenic Reagent and 60 µl Assay Buffer. After incubation at room temperature for 10 min, chromogenic complexes formed between calcium ions and o-cresolphthalein were detected at 575 nm with a microplate reader (Biotek, Synergy HT).

Phosphate assay

The phosphate concentration in the cell culture supernatant of ASMCs indirectly co-cultured with THP1 macrophages was determined using a malachite green calorimetric phosphate assay

(Abcam, #65622). Supernatants were applied in 1:500 dilution, incubated with phosphate reagent for 30 min and analyzed at 650 nm with a microplate reader (Biotek, Synergy HT).

Mineralization Assay

Alizarin Red S (Sigma Aldrich, #A5533) was used to visualize ectopic calcium deposition of cells undergoing osteoblastic-like differentiation for 21 d. After removing the cell culture media and washing three times with PBS, cells were fixed with 4% formaldehyde for 15 min at room temperature. The cells were washed three times with ddH$_2$O before staining the cells with freshly prepared 0.5% Alizarin Red S in ddH$_2$O for 20 min with gentle shaking. Unbound dye was removed by washing the cells at least three times with ddH$_2$O until the supernatant stayed clear, and the plate was allowed to dry at room temperature. For quantification of incorporated Alizarin Red S, the dye was extracted with 10% acetic acid and neutralized with 10% ammonium hydroxide followed by calorimetric detection at 405 nm with a microplate reader (Biotek, Synergy HT). An Alizarin Red S standard curve with concentrations ranging from 0.0313 mM to 2 mM was included to determine the amount of incorporated dye based on the absorption at 405 nm.

Bulk transcritpomic analysis

Public transcriptomic data from Tet2−/− and WT macrophages (Fuster et al., Science 2017) were reanalyzed. In the original study, RNA from unstimulated or LPS/IFN-γ–stimulated macrophages was hybridized to arrays, and tested for differential expression. In parallel, we performed bulk RNA-seq on human primary monocyte-derived macrophages treated with siNC or siTET2. Reads were trimmed with Trimmomatic v0.39 (Q < 15 in a 5-nt window, minimum length 15 nt), aligned to the Ensembl genome, and filtered with Picard to remove duplicates. Gene counts were obtained with featureCounts.

Statistical analysis

All biobank analyses were performed using Cox proportional hazards models with age as the time scale, adjusted for sex, ethnicity, type 2 diabetes, hypertension, and low density lipoprotein cholesterol. The age time scale approach used participants' age at blood draw as the entry time (time 1) and age at event occurrence or last follow-up as the exit time (time 2), with follow-up time calculated by adding the time elapsed since baseline to the baseline age. Statistical significance was assigned at alpha = 0.05. The analysis was >80% powered. All tests of significance are 2-sided. Comparison of demographic and follow-up data was performed using t tests for normal continuous data, Mann-Whitney U test for non-normal data, and χ^2 tests for categorical data. All analyses were performed using the statistical programming language R, version 4.0.2, with Cox regression models implemented using the survival package's coxph function with left-truncated, right-censored survival objects.(8)

In vitro cell culture and murine data were analyzed using Microsoft Excel or GraphPad Prism Software. Statistical significance was assessed by one-way ANOVA with Tukey's multiple comparison test, two-tailed unpaired Student's *t*-test. *P*-values <0.05 were considered statistically significant. For scRNA-seq analyses, statistical analysis of differential expression of genes was done with the FindMarkers function in the Seurat package. Multiple comparison statistical adjustment was made, and FDR corrected p-values of <0.05 were considered significant.

References

1. Vlasschaert C, et al. A practical approach to curate clonal hematopoiesis of indeterminate potential in human genetic data sets. *Blood*. 2023;141(18):2214–2223.
2. Jaiswal S, et al. Age-related clonal hematopoiesis associated with adverse outcomes. *N Engl J Med*. 2014;371(26):2488–98.

3. Jaiswal S, et al. Clonal Hematopoiesis and Risk of Atherosclerotic Cardiovascular Disease. *New England Journal of Medicine*. 2017;377(2):111–121.
4. Bick AG, et al. Genetic Interleukin 6 Signaling Deficiency Attenuates Cardiovascular Risk in Clonal Hematopoiesis. *Circulation*. 2020;124–131.
5. Abplanalp WT, et al. Single-cell RNA-sequencing reveals profound changes in circulating immune cells in patients with heart failure. *Cardiovasc Res*. 2021;117(2):484–494.
6. Hao Y, et al. Integrated analysis of multimodal single-cell data. *Cell*. 2021;184(13):3573-3587.e29.
7. Zhou Y, et al. Metascape provides a biologist-oriented resource for the analysis of systems-level datasets. *Nat Commun*. 2019;10(1):1523.
8. R Development Core Team. *R: A language and environment for statistical computing*. Vienna, Austria: R Foundation for Statistical Computing; 2020.
